# Clinical outcomes of myocardial infarction with non-obstructive coronary arteries presenting with diabetic ketoacidosis: a propensity score-matched analysis

**DOI:** 10.1186/s40001-023-01633-2

**Published:** 2024-01-08

**Authors:** Asif Ullah, Umar Khan, Shumaila Asif, Hafiz Muhammad Shafique, Talha Sajid, Jateesh Kumar, Waheed Akhtar, Syed Muhammad Jawad Zaidi, Jahanzeb Malik, Amin Mehmoodi

**Affiliations:** 1 Department of Cardiology, Khyber Medical Univerity Institute of Medical Sciences, Kohat, Pakistan; 2 Department of Pulmonology, University Hospital Kerry, Tralee, Ireland; 3 Department of Cardiology, Armed Forces Institute of Cardiology, Rawalpindi, Pakistan; 4 Department of Medicine, Shifa International Hospital, Islamabad, Pakistan; 5 Department of Medicine, Jinnah Sindh Medical University, Karachi, Pakistan; 6 Department of Cardiology, Abbas Institute of Medical Sciences, Muzaffarabad, Pakistan; 7 Department of Cardiovascular Medicine, Cardiovascular Analytics Group, Canterbury, UK; 8 Department of Medicine, Ibn e Seena Hospital, Kabul, Afghanistan

**Keywords:** Myocardial infarction, Diabetic ketoacidosis, Percutaneous coronary intervention, Coronary angiography

## Abstract

**Introduction and Objective:**

There is a paucity of data on patients with myocardial infarction with nonobstructive coronary arteries (MINOCA) and a decompensated diabetic state, diabetic ketoacidosis (DKA). Therefore, we aimed to investigate the outcomes of patients with MINOCA presenting with or without DKA.

**Methods:**

We conducted this retrospective propensity score-matched analysis from January 1, 2015, to December 4, 2022. The patients with a principal admission diagnosis of ST-Elevation MI (STEMI) and discharge labeled as MINOCA (ICD-10-CM code 121.9) with DKA were analyzed. We performed a comparative analysis for MINOCA with and without DKA before and after propensity score matching for primary and secondary endpoints.

**Results:**

Three thousand five hundred sixty-three patients were analyzed, and 1150 (32.27%) presented with DKA, while 2413 (67.72%) presented as non-DKA. The DKA cohort had over two-fold mortality (5.56% vs. 1.19%; p = 0.024), reinfarction (5.82% vs. 1.45%; p = 0.021), stroke (4.43% vs. 1.36%; p = 0.035), heart failure (6.89% vs. 2.11%; p = 0.033), and cardiogenic shock (6.43% vs. 1.78%; p = 0.025) in a propensity score-matched analysis. There was an increased graded risk of MINOCA with DM (RR (95% CI): 0.50 (0.36–0.86; p = 0.023), DKA (RR (95% CI): 0.46 (0.24–0.67; p = 0.001), and other cardiovascular (CV) risk factors.

**Conclusion:**

DKA complicates a portion of MINOCA and is associated with increased mortality and major adverse cardiovascular events (MACE).

**Supplementary Information:**

The online version contains supplementary material available at 10.1186/s40001-023-01633-2.

## Introduction

One of the significant risk factors for coronary artery disease (CAD) is diabetes mellitus (DM), and diabetics have a 50% higher risk of developing CAD than normal subjects [1]. Many investigations have shown that cardiovascular (CV) mortality and survival are affected by DM [2]. Mechanical complications, including ventricular septal defect, left ventricular aneurysm, congestive heart failure, cardiogenic shock, and conduction abnormalities, are more common in people with diabetes when compared to nondiabetics [3]. In addition, hyperglycemia is associated with adverse in-hospital outcomes and microvascular obstruction in acute coronary syndromes (ACS) [4].

However, there is a paucity of data on patients with myocardial infarction with nonobstructive coronary arteries (MINOCA) and a decompensated diabetic state, diabetic ketoacidosis (DKA). MINOCA is a condition with different causes, characterized by clinical evidence of myocardial infarction (MI) and angiographically normal or minimally obstructive (≤ 50% stenosis) coronary arteries [4]. MINOCA represents approximately 10% of acute coronary syndromes. DKA is a critical metabolic state, resulting in severe physiological derangements in the body [5]. It can lead to death if not addressed in an emergency. Insulin deficiency or resistance can cause counter-regulatory hormones (like glucagon) to increase in the bloodstream, causing a pro-inflammatory state in the presence of infection and other ongoing inflammation [6].

There's a shared set of risk factors that tie these conditions together. Both patients with DKA and MINOCA have common risk factors, such as diabetes, obesity, hypertension, and metabolic syndrome [7]. These overlapping risk factors can increase the likelihood of both conditions coexisting in an individual. Diagnostic challenges may emerge because the symptoms of DKA and MINOCA, such as chest pain and shortness of breath, can be similar. Consequently, healthcare providers must consider the potential interplay of these conditions, especially when evaluating patients with relevant symptoms and shared risk factors. Recognizing the indirect relationship between DKA and MINOCA is vital for comprehensive patient care and appropriate treatment decisions.

## Objectives

DKA is concomitantly seen among ACS patients. Therefore, we aimed to investigate the outcomes of patients with MINOCA presenting with or without DKA.

## Methods

Our team used the Abbas Institute of Medical Sciences patient database to derive patient-relevant information between January 1, 2015, and December 4, 2022. Abbas Institute of Medical Sciences permitted the use of the data for this study (Study ID # AIMS/22/039). All participants gave consent for their anonymized use of data. Patients > 18 years with a principal admission diagnosis of ST-Elevation MI (STEMI) (Internal Classification of Disease-Tenth Revision-Clinical Modification [ICD-10-CM] codes 121.0–122.9) were identified in the database by two investigators (J.M. and U.K.). The patients with routine coronary angiography (ICD-10-CM code 3,821,500) and discharge labeled as MINOCA (ICD-10-CM code 121.9) were analyzed. Nondiabetic patients were excluded, yielding a cohort of diabetic patients diagnosed with MINOCA. Those were then tabulated into patients with or without DKA. Similar to prior studies, we defined DKA as ICD-10-CM codes 250.10–250.13 [7].

We performed a comparative analysis for patients with MINOCA with and without DKA before and after propensity score matching for primary (in-hospital CV mortality) and secondary endpoints (in-hospital major adverse cardiovascular events [MACE], including re-infarct, heart failure [HF], and stroke). A propensity score-matching model was developed to control the potential confounding factors and reduce the effect of selection bias through logistic regression to form two matched groups for the comparative outcome analysis. Patients admitted with MINOCA with or without DKA were entered into the nearest 1:1 variable. A balanced propensity-matched model using a caliper of 0.01 without replacement was used to ensure homogeneous matching. Variables used in the propensity match model are tabulated in Additional file 1**: **Tables S1, S2.

Descriptive data were presented as frequency (n) and percentages (%) for categorical variables and as mean and standard deviation (SD) for continuous variables. Baseline characteristics were compared using the Chi-Square test, Fischer's exact test for categorical variables, and Student's t-test for continuous variables (normally distributed). The Wilk-Shapiro test was used for normality of distribution, and abnormally distributed variables were analyzed with the Mann-Whiney U test. The propensity score matching method was applied to address confounding and reduce bias in observational studies. Data on individuals, including their treatment status and covariates, were collected. Propensity scores, representing the likelihood of receiving treatment based on covariate values, were estimated using logistic regression. Matching criteria, such as nearest neighbor matching or caliper matching, were specified, and individuals from the treatment and control groups were matched based on their propensity scores, creating a balanced dataset. The balance of covariates was assessed post-matching to ensure that the treatment and control groups were comparable. We estimated the treatment effects from the matched dataset and conducted sensitivity analyses to evaluate the robustness of the results. Statistical inference was performed to draw conclusions and account for propensity score matching in the analysis. Matched categorical variables were presented as frequencies and percentages and compared using McNazar's test. Matched continuous data were presented as mean and SD and analyzed using the Student's paired-samples t-test. A p < 0.05 was considered statistically significant. All data were analyzed using the Statistical Package for Social Sciences (SPSS) version 26 (IBM Corp., Armonk, NY, USA).

## Results

A total of 80,364 patients admitted with ACS between January 1, 2015, and December 4, 2022, were included in this study. Of those, 3,563 were diagnosed with MINOCA and had diabetes. Of these, 1,150 (32.27%) presented with DKA, and 2,413 (67.72%) presented as non-DKA patients. Patients with DKA were younger (63 ± 12 vs. 64 ± 9; p = 0.036) and had a distinctive risk profile compared with non-DKA patients, characterized by a higher incidence of anemia (20% vs. 14.81%; p = 0.026), congestive heart failure (4.95% vs. 1.95%; p = 0.015), cardiogenic shock (19.04% vs. 9.64%; p < 0.001), smoking (23.39% vs. 16.91%; p = 0.011), coagulopathy (8.17% vs. 3.37%; p = 0.025), and hyperthyroid (10.34% vs. 3.66%; p < 0.001). Baseline characteristics are tabulated in Table 1. Intravenous thrombolysis (42.79% vs. 38%; p = 0.034) was performed more in patients without DKA, and percutaneous coronary intervention (PCI) was performed in a relatively low percentage in both groups (4.43% vs. 5.65%; p = 0.237). The treatment pattern is shown in Table 1.

**Table 1:** Baseline characteristics

Baseline characteristics (Total = 80,364)	DKA (n = 1,150)	Non-DKA (n = 2,413)	P-value
Age; mean ± SD	63 ± 12	64 ± 9	0.036
Female	503 (43.73%)	970 (40.24%)	0.078
Hypertension	582 (50.6%)	1,002 (41.52%)	0.044
Dyslipidemia	485 (42.17%)	1,187 (49.2%)	0.012
Chronic lung disease	177 (15.39%)	380 (15.76%)	0.956
Atrial fibrillation	154 (13.39%)	367 (15.22%)	0.093
Anemia	230 (20%)	357 (14.81%)	0.026
Congestive heart failure	57 (4.95%)	47 (1.95%)	0.015
Cardiogenic shock	219 (19.04%)	232 (9.64%)	< 0.001
Peripheral vascular disease	81 (7%)	300 (12.44%)	0.023
Prior stroke	45 (3.91%)	81 (3.37%)	0.935
Chronic kidney disease	207 (18%)	461 (19.12%)	0.682
Liver disease	21 (1.8%)	40 (1.65%)	0.824
Smoking	269 (23.39%)	408 (16.91%)	0.011
Drug abuse	39 (3.39%)	44 (1.84%)	0.067
Coagulopathy	94 (8.17%)	81 (3.37%)	0.025
Hyperthyroid	119 (10.34%)	88 (3.66%)	< 0.001
Treatment patterns			
Percutaneous coronary intervention	65 (5.65%)	106 (4.43%)	0.237
IV thrombolysis	437 (38%)	1,032 (42.79%)	0.034

Compared to patients without DKA, the DKA cohort had over two-fold mortality (5.91% vs. 1.02%; p = 0.012), reinfarction (6.08% vs. 1.27%; p = 0.019), stroke (3.73% vs. 0.91%; p = 0.038), heart failure (6.6% vs. 1.78%; p = 0.013), cardiogenic shock (6.43% vs. 1.69%; p = 0.017), acute kidney injury (23.91% vs. 5.47%; p < 0.001), sepsis (7.3% vs. 1.36%; p = 0.007), urinary tract infection (15.13% vs. 2.36%; p < 0.001), and pneumonia (7.04% vs. 2.36%; p = 0.005). They also had more extended hospitalizations (9 ± 6 days vs. 5 ± 4 days; p < 0.001) and more mechanical ventilation (8.86% vs. 1.82%; p = 0.006) (Table 2). Following propensity-score matching, the baseline characteristics of patients with MINOCA with and without DKA were well matched (Additional file 1**: **Tables S1, S2, and S3). The presence of DKA was associated with low referral rates for coronary angiography (34.21% vs. 53.72%; p < 0.001) (Additional file 1: Table S3). In this propensity-matched cohort, DKA was associated with a higher incidence of primary and secondary outcomes (Table 2). Mortality (5.56% vs. 1.19%; p = 0.024), reinfarction (5.82% vs. 1.45%; p = 0.021), stroke (4.43% vs. 1.36%; p = 0.035), heart failure (6.89% vs. 2.11%; p = 0.033), cardiogenic shock (6.43% vs. 1.78%; p = 0.025), and sepsis (8% vs. 1.45%; p = 0.005) were significantly higher with DKA (Table 2).

**Table 2:** Clinical outcomes

Clinical outcomes	Unmatched cohort			Matched cohort		
	DKA (n = 1,150)	Non-DKA (n = 2413)	P-value	DKA (n = 1150)	Non-DKA (n = 2413)	P-value
Mortality	68 (5.91%)	25 (1.02%)	0.012	64 (5.56%)	29 (1.19%)	0.024
Reinfarction	70 (6.08%)	31 (1.27%)	0.019	67 (5.82%)	35 (1.45%)	0.021
Stroke	43 (3.73%)	22 (0.91%)	0.038	51 (4.43%)	33 (1.36%)	0.035
Heart failure	76 (6.6%)	43 (1.78%)	0.013	79 (6.89%)	51 (2.11%)	0.033
Cardiogenic shock	74 (6.43%)	41 (1.69%)	0.017	74 (6.43%)	43 (1.78%)	0.025
Acute kidney injury	275 (23.91%)	132 (5.47%)	< 0.001	289 (25.13%)	108 (4.47%)	< 0.001
Sepsis	84 (7.3%)	33 (1.36%)	0.007	92 (8%)	35 (1.45%)	0.005
Mechanical ventilation	102 (8.86%)	44 (1.82%)	0.006	109 (9.47%)	52 (2.15%)	0.003
Urinary tract infection	174 (15.13%)	57 (2.36%)	< 0.001	195 (16.95%)	63 (2.61%)	< 0.001
Pneumonia	81 (7.04%)	57 (2.36%)	0.005	83 (7.21%)	56 (2.32%)	0.004
Length of stay; mean ± SD	9 ± 6	5 ± 4	< 0.001	9 ± 5	5 ± 5	< 0.001

Given the patients in the DKA group were likely to have CAD, the impact of MINOCA was seen through graded risk (relative risk [RR]) in various comorbidities associated with it (Table 3). There was an increased graded risk of MINOCA with DM (RR(95% CI): 0.50 (0.36–0.86); p = 0.023), DKA (RR(95% CI): 0.46 (0.24–0.67); p = 0.001), peripheral arterial disease (RR(95% CI): 0.75 (0.57–0.96); p = 0.025), renal disease (RR(95% CI): 0.52 (0.35–0.83); p = 0.04), and smoking (RR(95% CI): 0.76 (0.37–0.96); p = 0.006) (Table 3).

**Table 3:** Graded risk of MINOCA with DKA and other CV risk factors

Variables	Multivariate model; relative risk (95% CI)	p-value	Variables	Multivariate model; relative risk (95% CI)	p-value
MINOCA + DM	0.50 (0.36–0.86)	0.023	No MINOCA + DM	0.31 (0.23–0.57)	0.005
MINOCA + DKA	0.46 (0.24–0.67)	0.001	No MINOCA + DKA	0.51 (0.15–0.93)	0.014
MINOCA + PAD	0.75 (0.57–0.96)	0.025	No MINOCA + PAD	2.15 (1.27–2.64)	0.084
MINOCA + renal disease	0.52 (0.35–0.83)	0.04	No MINOCA + Renal disease	1.45 (1.12–1.68)	0.063
MINOCA + Smoking	0.76 (0.37–0.96)	0.006	No MINOCA + Smoking	0.57 (0.65–0.81)	0.027

## Discussion

The main findings of this investigation are: (i) DKA occurs in 1.43% of people with diabetes admitted with ACS; (ii) these patients have a higher prevalence of HF, cardiogenic shock, and coagulopathy; (iii) patients with DKA were less likely to undergo coronary angiography and revascularization; (iv) overall MACE was higher in patients with DKA; (v) MINOCA can also present as a DKA state and confer a higher graded risk of DKA; have a higher incidence of specific comorbidities; and a lengthy hospital stay.

**Fig. 1:**
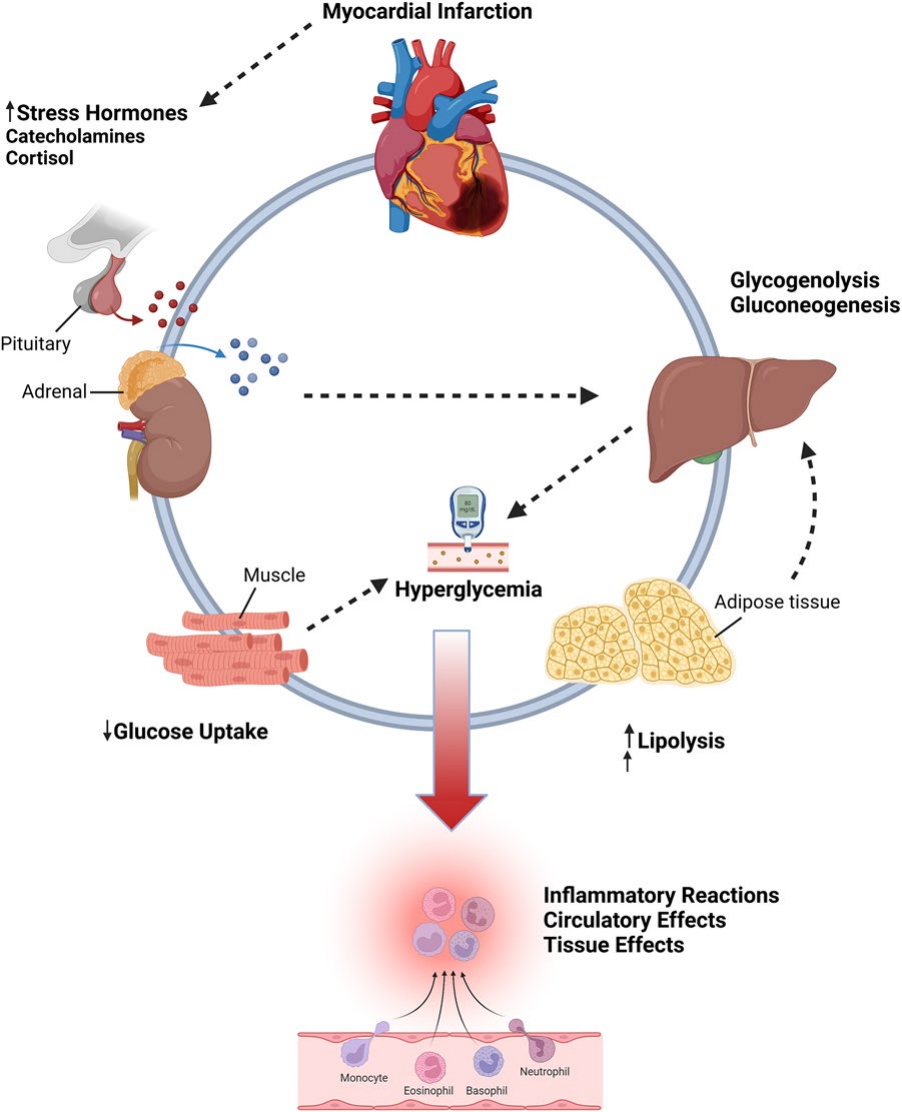
Cardiovascular effects of hyperglycemia during the acute phase of myocardial infarction

DKA occurs typically during physiologically stressful states, such as in patients with severe infections, sepsis, major burns, multiple traumas, and ACS. Stress hyperglycemia occurs due to sympathetic nervous activation and the hypothalamic-pituitary axis with concomitant production of cortisol and catecholamines that trigger glycogenolysis, gluconeogenesis, and lipolysis [8]. Although the potential mechanism between DKA and mortality in patients with ACS is unknown, there is evidence that using insulin to lower blood glucose levels during ACS can reduce mortality. This suggests that DKA is not an epiphenomenon of stress responses mediated by stress hormones [9]. DKA has been demonstrated to increase the release of pro-inflammatory cytokines, decrease coronary endothelial function, produce reactive oxygen species, and stimulate platelet aggregation (Fig. 1) [9]. Furthermore, DKA lengthens QT intervals, decreases preconditioning of myocardium to ischemia, and increases no-reflow/myocardial blush [10]. It can also potentiate insulin resistance, causing impaired glucose utilization and potentially exacerbating ischemia through elevated oxygen use [11].

MINOCA is a syndrome caused by multiple pathophysiological mechanisms, and due to nonobstructive coronaries, it is often misdiagnosed and not given full attention. Various reports show the prevalence of MINOCA with ACS between 1 and 15% [12–16]. To evaluate MINOCA, the European Society of Cardiology published a working paper 2018 that included MINOCA as a type of myocardial infarction (MI) in the Fourth Universal Definition of Myocardial Infarction [17]. MINOCA is caused by epicardial vascular causes (coronary plaque disruption, coronary artery spasm, and spontaneous coronary dissection) and microvascular causes (coronary microvascular dysfunction, coronary thromboembolism, and microcirculation spasm) [14]. The mechanisms responsible for DKA and MINOCA overlap to some extent; therefore, a decompensated hyperglycemic state can produce MI without epicardial coronary obstruction. The two altered metabolic states can also change the functional properties of myocytes and platelets [18]. An altered platelet metabolism can contribute to developing endothelial dysfunction, atherothrombotic complications, and microvascular obstruction, causing MINOCA [14].

Prior studies have shown an association between hyperglycemic states and ST-Elevation MI (STEMI) in diabetic and nondiabetic patients [19, 20]. Furthermore, it has been demonstrated to have a worse prognosis for short- and long-term outcomes [21]. However, DKA and its impact on MINOCA have yet to be previously investigated. Issa et al. have shown that DKA complicates 1.5% of STEMI admissions and is associated with lower revascularization rates and coronary angiography referrals [19]. Similarly, our study demonstrated a slightly lower incidence (1.43%) of DKA in patients labeled as ACS.

Furthermore, 32.27% of patients with MINOCA presented with DKA in our study, showing a relationship between the two pathologies. The higher prevalence of CAD and worse baseline presentation (evident with the higher incidence of HF and cardiogenic shock) in the DKA population is not surprising. Speculatively, these patients may have been constituted in the cohort with worse chronic glycemic control, elevated symptoms due to diabetic complications, late presentation, and possibly more considerable cardiac injury [22].

The relationship between DKA and worse outcomes in patients with MINOCA is rather complex. We analyzed the graded risk of DKA in MINOCA, demonstrating a strong association between the two entities. Several studies have shown worse STEMI outcomes in patients with DKA due to larger infarct sizes and the no-reflow phenomenon [10, 23, 24]. In contrast, the occurrence of DKA during ACS admission can also cause a worse prognosis for patients. However, we did not analyze it in our cohort, and further studies are needed to identify the underlying mechanisms associated with hyperglycemic states. Patients in our cohort were less likely to receive a coronary angiogram and revascularization. Our group needed to ascertain the reasons for less invasive practices in this study.

In our opinion, patients presenting with DKA and labeled as MINOCA after coronary imaging should be managed rigorously for diabetic control, and thromboprophylaxis should be given with dual antiplatelets for at least three months.

Our study had several limitations: (i) the observational nature of the study might have produced inherent limitations of cause and effect; (ii) angiographic data and peri-procedural complications, hemodynamics, and medication use are not available in our data; (iii) data on blood glucose levels and insulin use, as well as the timing of DKA diagnoses, were not captured in the database; and (iv) preadmission control of blood sugar and anti-diabetic medications were not included in the data set. Despite differences in the baseline profiles of the two groups, our propensity score matching should minimize the risk associated with unmeasured confounders in comparative analysis.

## Conclusion

DKA complicates 1.43% of ACS and 32.27% of patients with MINOCA in diabetic patients. It is associated with increased MACE and in-hospital mortality and a negative differential impact on the length of hospital stay. Furthermore, an increased graded risk of DKA is seen in the MINOCA cohort; even after propensity score matching, the MACE remains high in the DKA group. Further studies are needed to identify the pathophysiological mechanism behind this association and to provide a preventive strategy to mitigate the increased mortality in these patients.

### Supplementary Information

**Additional file 1: Table S1.** Variables used for propensity score matching. **Table S2.** Baseline characteristics of the propensity matched group. **Table S3.** Treatment patterns in propensity matched group.

## Data Availability

Data is available from corresponding author upon reasonable request.
