# Supplementary material for: Clinical outcomes of myocardial infarction with non-obstructive coronary arteries presenting with diabetic ketoacidosis: a propensity score-matched analysis
Source: Eur J Med Res. 2024 Jan 8;29:36. doi: 10.1186/s40001-023-01633-2 (PMC10773043; doi:10.1186/s40001-023-01633-2)
Supplement: Supplementary file 1 — Additional file 1: Table S1. Variables used for propensity score matching. Table S2. Baseline characteristics of the propensity matched group. Table S3. Treatment patterns in propensity matched group. [file 40001_2023_1633_MOESM1_ESM.docx]

| Age | Peripheral vascular disease |
| --- | --- |
| Female | Prior stroke |
| Hypertension | Chronic kidney disease |
| Dyslipidemia | Liver disease |
| Chronic lung disease | Smoking |
| Atrial fibrillation | Drug abuse |
| Anemia | Coagulopathy |
| Congestive heart failure | Hyperthyroid |
| Cardiogenic shock | Conduction abnormality |
| Length of stay | Graded risk of DKA |

**Additional file 1: Table S1.** Variables used for propensity score matching

| **Baseline characteristics (Total=80,364)** | **DKA (n=1,150)** | **Non-DKA (n=2,413)** | **P-value** |
| --- | --- | --- | --- |
| Age; mean ± SD | 63 ± 12 | 64 ± 9 | 0.865 |
| Female | 43.73% | 42.24% | 0.478 |
| Hypertension | 50.6% | 48.07% | 0.594 |
| Dyslipidemia | 42.17% | 49.2% | 0.712 |
| Chronic lung disease | 15.39% | 15.76% | 0.956 |
| Atrial fibrillation | 13.39% | 15.22% | 0.393 |
| Anemia | 20% | 14.81% | 0.226 |
| Congestive heart failure | 4.95% | 4.34% | 0.965 |
| Cardiogenic shock | 19.04% | 18.64% | 0.541 |
| Peripheral vascular disease | 11.4% | 12.44% | 0.423 |
| Prior stroke | 3.91% | 3.37% | 0.935 |
| Chronic kidney disease | 18% | 19.12% | 0.682 |
| Liver disease | 1.8% | 1.65% | 0.824 |
| Smoking | 23.39% | 23.91% | 0.315 |
| Drug abuse | 3.39% | 2.84% | 0.267 |
| Coagulopathy | 8.17% | 3.37% | 0.625 |
| Hyperthyroid | 10.34% | 9.66% | 0.321 |

**Additional file 1: Table S2.** Baseline characteristics of the propensity matched group

| **Treatment** | **DKA (n=1,150)** | **Non-DKA (n=2,413)** | **P-value** |
| --- | --- | --- | --- |
| Coronary angiography | 34.21% | 53.72% | <0.001 |
| Percutaneous coronary intervention | 5.65% | 5.43% | 0.237 |
| IV thrombolysis | 41.64% | 42.79% | 0.434 |

**Additional file 1: Table S3.** Treatment patterns in propensity matched group
